# Higher serum concentrations of vimentin and DAKP1 are associated with aggressive breast tumour phenotypes in Ghanaian women

**DOI:** 10.1186/s40364-017-0100-0

**Published:** 2017-06-09

**Authors:** Benjamin Arko-Boham, Justice Tanihu Lomotey, Emmanuel Nomo Tetteh, Emmanuel Ayitey Tagoe, Nii Ayite Aryee, Ewurama Ampadu Owusu, Isaac Okai, Richard Michael Blay, Joe-Nat Clegg-Lamptey

**Affiliations:** 1 Department of Medical Laboratory Sciences, School of Biomedical and Allied Health Sciences, College of Health Sciences, University of Ghana, Korle-Bu, Accra, Ghana; 2 West African Centre for Cell Biology of Infectious Pathogens, University of Ghana, Legon, Accra, Ghana; 3 Department of Medical Biochemistry, School of Biomedical and Allied Health Sciences, College of Health Sciences, University of Ghana, Accra, Ghana; 4 Centre of Tropical Medicine and Travel Medicine, Department of Infectious Diseases, Division of Internal Medicine, Academic Medical Centre, University of Amsterdam, Amsterdam, The Netherlands; 5 Department of Anatomy, School of Medical Sciences, Kwame Nkrumah University of Science and Technology, PMB, Kumasi, Ghana; 6 Department of Anatomy, School of Biomedical and Allied Health Sciences, University of Ghana, Accra, Ghana; 7 Department of Surgery, School of Medicine and Dentistry, College of Health Sciences, University of Ghana, Accra, Ghana; 8 Department of Surgery, Korle-Bu Teaching Hospital, Accra, Ghana

**Keywords:** Vimentin, Death-Associated Protein Kinase 1(DAPK1), Serum concentration, Breast cancer, Aggression, Ghanaians

## Abstract

**Background:**

Breast cancer, the most commonly diagnosed cancer among women and leading cause of cancer-related deaths worldwide, exhibits aggressive behavior in indigenous African women evidenced by high histologic grade tumours with low hormone receptor positivity. Aggressive breast cancers grow quickly, easily metastasize and recur and often have unfavourable outcomes. The current study investigated candidate genes that may regulate tumour aggression in Ghanaian women. We hypothesize that increased expression and function of certain genes other than the widely-held view attributing breast cancer aggression in African populations to their younger population age may be responsible for the aggressive nature of tumours.

**Methods:**

Employing ELISA, we assayed for vimentin and death-associated protein kinase 1 (DAPK1) from thawed archived (stored at -80 °C) serum samples obtained from 40 clinically confirmed Ghanaian breast cancer patients and 40 apparently healthy controls. Patients’ clinical records and tumour parameters matching the samples were retrieved from the database of the hospital. ANOVA was used to compare means of serum protein concentration among groups while Chi-square analysis was used for the categorical data sets with *p*-value ≤0.05 considered significant. Multiple logistic regression analysis was conducted to determine the association between protein concentration and tumour parameters.

**Results:**

Of the 80 samples, 27 (33.8%) and 53 (66.2%) were from young (<35 years) and old (≥35 years), respectively. Vimentin and DAPK1 concentration were higher in patients than controls with higher levels in “young” age group than “old” age group. Vimentin concentration was highest in grade 3 tumours followed by grade 2 and 1 but that for DAPK1 was not significant. For vimentin, tumour area strongly correlated with tumour grade (*r* = 0.696, *p* < 0.05) but weakly correlated with tumour stage (*r* = 0.420, *p* < 0.05). Patient’s age correlated with DAPK1 concentration (*r* = 0.393, *p* < 0.05). DAPK1 serum levels weakly correlated with cancer duration (*r* = 0.098, *p* = 0.27) and tumour size (*r* = 0.40, *p* < 0.05).

**Conclusion:**

Serum concentration of Vimentin and DAPK1 are elevated in Ghanaian breast cancer patients. This may be partly responsible for aggressive nature of the disease among the population. Vimentin and DAPK1 should be explored further as potential breast cancer biomarkers in Africans.

## Background

Breast cancer is a malignant tumour that starts in the cells of the breast with the potential of metastasis. It remains the most common malignancy in women across the world [1]. In 2012, an estimated 1.7 million women were diagnosed with breast cancer worldwide and there were 6.3 million living with the disease in the previous 5 years [1]. Breast cancer is one of the most common causes of cancer deaths among women with 522,000 deaths in 2012 [2, 3]. In Western Europe, breast cancer incidence has reached more than 90 new cases per 100,000 women annually compared with 30 per 100,000 in Eastern Africa [2]. According to Jemal et al. [4], breast cancer is the most prevalent cancer malignancy and the leading cause of cancer-related mortality in women in developed countries. In a survey, an estimated 232,670 women were expected to be diagnosed with breast cancer, and 40,000 deaths expected in 2014 [5]. In Europe in 2012, an estimated 463,800 new breast cancer cases and breast cancer-related deaths of 131,200 were recorded [6].

The disease is also the leading cause of cancer-related deaths among West African women with over 30,000 new cases between 2008 and 2012 with more than 16,000 deaths [7]. Caucasians have a higher incidence of breast cancer than people of Black Africans descent but the mortality rate for women of Black African descent surpasses that of Caucasians thus presenting a poorer survival rate for women of Black African descent [7, 8]. There are contrasting characteristics of breast cancer diagnosed in African women as compared to Caucasians such as high grade, advanced stage and negative hormone receptor status [7]. African-American women have greater likelihood of having more aggressive breast cancer pathology such as inflammatory, medullary or papillary carcinoma and less are likely to have tubular or lobular carcinoma than Caucasians [9]. A number of studies have proposed there are epidemiological differences between breast cancers among women in Europe and Africa. Risk factors such as age at menopause, oral contraceptive use, cigarette smoking and family history of breast cancer have been shown to have different significance to breast cancer development among women in Africa and Europe [10].

Breast Cancer deaths are disproportionately higher compared to incidence in less developed countries including Ghana [11]. It is disturbing that breast cancer constitutes 15.4% of all female cancer deaths [12] and 12.8% of all hospital admissions in Ghana [13]. In Ghana, breast cancer, which was believed primarily to be a disease of the elderly women, seems to occur in relatively younger women [14, 15]. Between the year 2013 and 2014, 79% of new cases and 88% deaths to breast cancer in non-Hispanic white females in the United States of America occurred in women aged 50 years and above [16]. However, women in Africa present a different picture, where majority of breast cancer cases occur in relatively younger women. A 10 year review of pathology specimens in Ghana revealed that the disease occurred more commonly between ages 36–45 years with less than 5% in women aged above 50 years [17, 18]. According to Bowen and colleagues [19] the age at which African women present breast cancer at the hospital is low and the stage late, compared to their European counterparts. Whereas African women generally present at hospitals at relatively lower age and with advanced tumour stage, mostly stage III, their European counterparts generally present at relatively less advanced age and at early stages, mostly stage I of the disease. These findings have been interpreted to suggest that breast cancer in African women and in particular sub-Saharan African women is more aggressive than in Caucasians. However, other researchers have reports to the contrary, suggesting that the cause of the aggressive forms of breast cancer diagnosed in African women is not due to genes peculiar to the African race but rather due to epidemiological risk factors such as late stage and early age at presentation [7]. The relatively poor prognosis of breast cancer in sub-Saharan Africa has been attributed in part to the late stage at presentation (i.e. large tumour sizes, metastatic disease, node positive tumours) as well as the biological characteristics of the tumours (high grade, high triple negative/basal-type tumours). Little is known about the biomarkers that can predict breast cancer aggressiveness that may be peculiar to women of Black African descent.

This work reports on two proteins, vimentin and Death Associated Protein Kinase 1 (DAPK1) as targets for predicting tumour aggression in breast cancer development and progression. Vimentin is a 57 kDa protein and one of the most highly conserved proteins of the type III intermediate filament protein family with its expression starting on embryonic day 8.5 during murine development [20]. In human adults, vimentin expression is restricted to the connective tissue mesenchymal cells of the central nervous system and muscles [21]. Vimentin is also known to maintain cellular integrity and provide resistance against stress. Vimentin, like all intermediate-sized filaments (IF) is largely an insoluble intracellular protein polymer structure but recent reports have suggested that vimentin displays dynamic and complex expression as both soluble and polymeric proteins [22, 23]. The soluble forms of vimentin in human vascular endothelial cells cross the cell membrane and end up in the blood stream increasing serum concentration of the protein [24, 25]. Its expression has been reported in various epithelial cancers including prostate cancer, CNS tumours, lung and breast cancers [26]. Vimentin expression has been shown to be elevated in several aggressive breast cancer cell lines [27] and this increased expression positively correlates with increased migration and invasion of breast cancer cells [27, 28]. Due to its elevated expression in most cancers, it is very essential to determine the role of vimentin in cancers including that of the breast.

The Death-Associate Protein kinase (DAPK) family is a novel group of protein kinases which have the capacity to mediate apoptosis through their catalytic activities. The family is made up of three closely related serine/threonine kinases [Death-Associated Protein kinase (DAPK; DAPK1), DAPK-1 related protein 1 (DRP-1; DAPK-2) and Zipper interacting protein (ZIPK; DAPK- 3)], which display a high degree of homology in their catalytic domains [29]. DAPK 1 is traditionally known to induce the death of cells. However, studies have shown its importance in the rapid growth of breast cancers [30]. DAPK1 expression has been found to be elevated in certain types of breast cancers which are typically more aggressive and have poor prognosis. Its expression has also been shown to be increased in breast cancers with mutations in the TP53 protein [30]. This mutation is abundant in breast cancers which are negative for oestrogen hormone receptor [29, 30] but DAPK1 and Vimentin expression profiles in breast cancer in African women are not known.

## Methods

### Study design and population

The study was a retrospective case control study conducted on 80 archived (stored at -80 °C) serum samples collected from 40 breast cancer patients and 40 apparently healthy control subjects. Archived samples used in this study were previously obtained and stored sera of patients clinically diagnosed with breast cancer at the Department of Surgery of the Korle-Bu Teaching and control subjects from the Avenor Community in Accra, Ghana. Ethical approval was given by the Ethical and Protocol Review Committee of the School of Biomedical and Allied Health Sciences of the University of Ghana.

### Sample collection and clinical data of participants

Whole blood samples (5 ml each) were collected from consented participants into EDTA anti-coagulated tubes. Blood samples were centrifuged at 3000 rpm for 5 min to separate sera from formed elements. The sera were then collected into Eppendorf tubes and stored at -80 °C until use.

Pertinent demographic data on the participants were obtained. Data collected from breast cancer patients included the biopsy numbers (case numbers), age, duration of cancer, tumour sizes and histological grades at the time of diagnosis. These were retrieved from the patients’ folders from the Hospital’s database.

### ELISA assay

Stored sera were retrieved from -80 °C and slowly thawed at room temperature for 20 min. Sandwich- Enzyme Linked Immunosorbent Assay (ELISA) technique was used to determine the concentrations of vimentin and DAPK1 in sera of participants. Serum samples and standards were added into the appropriate micro ELISA plate wells pre-coated with anti-vimentin and anti-DAPK1 antibodies (Sunlong Biotech, PR China). Horseradish Peroxidase (HRP)-conjugated antibody specific for vimentin and DAPK1, were added to the wells and incubated for 20 min. Free unbound components were washed away. The TMB substrate solution was added to each well and incubated for 10 min. Subsequently, a stop solution was added to each well turning the blue colouration to yellow. The optical density (OD) was measured spectrophotometrically at a wavelength of 450 nm. Vimentin and DAPK1 concentrations were determined from standard curves.

### Statistics and data analysis

Statistical analysis was performed using SPSS 23 software. Descriptive statistics was used to summarize the data. Student t-test and one way ANOVA were used to compare means of grouped quantitative data. Comparison between categorical data was done using Chi-square test. Pearson correlation was used to determine the association between the clinical parameters. In all analyses, *p*-value ≤0.05 was considered statistically significant.

## Results

### Demographics of study participants

Out of the total number (80) of participants considered, 27 were young participants aged below 35 years representing 33.8% of the study population and 53 older participants aged between 35 and 75 years accounting for 66.2% of the total participants. The test patients were relatively more advanced in age compared to the control group (*p* = 0.006) as shown in Table 1. Out of the 40 breast cancer subjects considered, 7 were young patients aged <35 years representing 17.5% of the total and 33 older patients aged ≥35 years accounting for 82.5% of the total participants. Majority of the study subjects were aged above 35 (82.5%), illustrating that a greater percentage of older women were diagnosed with breast cancer within the period of this study as compared to the comparator age group. Also presented in Table 1 is the occupational profile of participants.

**Table 1:** Age distribution of study participants

Age (years)	Breast cancer participants (*n*)	Controls (*n*)	Total	Percentage (%)	*p*-value
Young (<35) Old (≥35)	7 33	20 20	27 53	33.7 66.3	
Mean age	46.3 ± 9.4	40.1 ± 14.4			0.006
Total			80	100	
Occupation					
Traders Civil servants Unskilled labour Unemployed Others Total	21 4 4 3 8 40	24 2 7 2 5 40	45 6 11 5 13 80	56.3 7.5 13.8 6.3 16.3 100	0.280

*n* number. Mean age is presented as mean ± SD. Comparison between parameters were determined by chi-square, t-test. *P*-value <0.05 is considered significant

### Tumour characteristics of breast cancer participants

The pathological reports of the 40 breast cancer patients included in the study are summarized in Table 2. Patients less than 35 years of age were 7(17.5%) and the patients 35 years of age and above were 33(82.5%). The tumour stages were categorized as 1, 2, 3, 4, x, where x is used to define tumours which could not be staged. The respective frequencies and percentages have been recorded in Table 2. The tumours were categorized into grades 1, 2, 3 with their respective frequencies and percentages. The mean breast tumour area (size) was 1.94 ± 1.65 cm^2^. The tumour areas were categorized on scale of 0.0–1.0, 1.1–2.0, 2.1–3.0, 3.1–4.0, 4.1–5.0, 5.1–6.0 cm^2^. The respective frequencies and tumour stages are shown in Table 2.

**Table 2:** Tumour characteristics of study participants

Tumour characteristics	All participants (*n* = 40) (%)	Participants <35 years (*n* = 7) (17.5%)	Participants ≥35 years (*n* = 33) (82.5%)
Primary tumour (n)			
T1 T2 T3 T4 Tx	0 (0.00) 6 (15) 11 (27.5) 16 (40) 7 (17.5)	0 (0.00) 2 (28.6) 2 (28.6) 2 (28.6) 1 (14.3)	0 (0.00) 4 (12.1) 9 (27.3) 14 (42.4) 6 (18.2)
Tumour area (cm^2^)			
0.0–1.0 1.1–2.0 2.1–3.0 3.1–4.0 4.1–5.0 5.1–6.0	14 (35) 8 (20) 11 (27.5) 2 (5) 2 (5) 2 (5)	4 (57.1) 2 (28.6) 0 (0.00) 0 (0.00) 1 (14.3) 0 (0.00)	10 (30.3) 6 (18.2) 11 (33.3) 2 (6.1) 1 (3.0) 2 (6.1)

### Vimentin and DAPK1 concentration pattern in serum samples

**Fig. 1:**
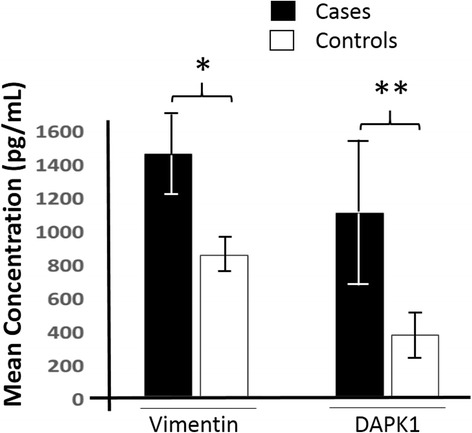
A bar graph comparing vimentin and DAPK1 concentrations in serum samples between breast cancer patients and apparently healthy controls. The data are presented as the mean ± SE; *n* = 3. * *p* = 0.001; **p = 0.02

All eighty (80) serum samples were analyzed and the concentrations of vimentin and DAPK1 were determined. The concentrations of vimentin (*p* = 0.001) and DAPK1 (*p* = 0.02) were observed to be significantly higher in the serum samples of breast cancer patients than those of the apparently healthy controls as shown in Fig. 1. Whereas vimentin concentration averaged 1462.63 ± 175.23 ng/ml in breast cancer patients and 851.29 ± 109.04 ng/ml in controls, DAPK1 concentration averaged 1105.75 ± 396.56 ng/ml and 371.09 ± 119.82 ng/ml in breast cancer patients and controls, respectively.

### Vimentin and DAPK1 serum concentration pattern among age groups

**Fig. 2:**
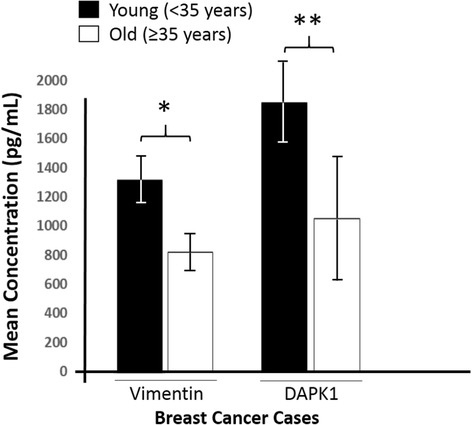
Vimentin and DAPK1 serum concentration pattern in breast cancer patients with respect to age categorization. “Young” breast cancer patients (<35 years) had elevated serum levels of the two proteins than the “old” patients (≥35 years). The data are presented as the mean ± SD; *n* = 3. *p = 0.01; **p = 0.03

Breast cancer patients were grouped into two main categories using the World Health Organization’s (WHO) reference intervals. Subjects below 35 year of age are categorized as “Young” and those 35 years and above as “Old” (Table 1). Both vimentin and DAPK1 serum concentratione were significantly elevated in the “young” group than the “old” group with *p-values* of 0.01 and 0.03 respectively (Fig. 2). The mean vimentin concentration was, respectively, 1289 ± 118 ng/ml and 802 ± 92 ng/ml in the “young” and “old” populations; and that for DAPK1, 1801 ± 215 ng/ml and 1025 ± 408 ng/ml, respectively, for the “young” and “old” populations. However, the serum concentration of the proteins among the apparently healthy controls with respect to the age categorization was insignificant (*p* = 1.00). The average duration of breast cancer for the patients considered in this study was 8 months. However the distribution of cancer duration was the same across the age categories, thus the difference observed between the durations of “young” and “old” patients was not statistically significant (*p* = 0.146).

### Vimentin and DAPK1 serum concentration pattern among tumour grades

**Fig. 3:**
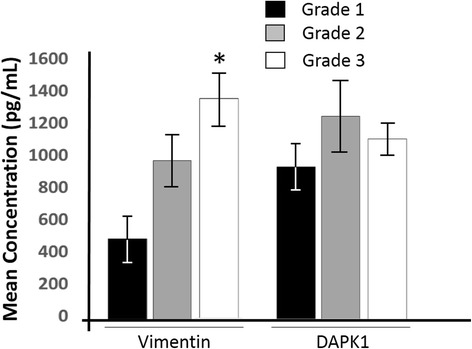
Vimentin and DAPK1 serum concentration pattern among different tumour grades. The decreasing order of vimentin concentration among tumour grades was 1350 ± 250 pg/ml, 990 + 200 pg/ml and 490 ± 120 pg/ml respectively for grade 3, grade 2 and grade 1 with significant difference in mean concentration among the tumour grades. For DAPK1 however, there was insignificant difference in the mean concentration among tumour grades (Grade 1 = 930 ± 150 pg/ml, Grade 2 = 1230 ± 245 pg/ml, and Grade 3 = 1090 ± 110 pg/ml) (*p* = 0.226). The data are presented as the mean ± SD; *n* = 3. *p = 0.019

The serum concentrations of vimentin and DAPK1 were assessed in the serum samples of the breast cancer patients with respect to tumour grading. For vimentin, it was observed that the mean serum concentration in tumour grade 3 was highest (1350 ± 250 pg/ml) followed by grade 2 (990 + 200 pg/ml) and grade 1 (490 ± 120 pg/ml) (Fig. 3). DAPK1 levels among Grade 1, Grade 2 and Grade 3 were 930 ± 150 pg/ml, 1230 ± 245 pg/ml and 1090 ± 110 pg/ml, respectively. However, the association between DAPK1 levels and the tumour grades was not statistically significant (*p* = 0.562).

### Correlation between tumour parameters and vimentin and DAPK1 concentration

Patient’s age and tumour parameters including stage, tumour duration and tumour area (size) were correlated with vimentin and DAPK1 serum concentration using multiple regression analysis and the results summarized in Table 3. For vimentin, serum levels strongly and positively correlated tumour area (*r* = 0.621, *p* < 0.05) and tumour grade (*r* = 0.696, *p* < 0.05) but showed a weak correlation with tumour stage (*r* = 0.420, *p* < 0.05). With DAPK1, patient’s age weakly but positively correlated with the concentration of DAPK1 (*r* = 0.393, *p* < 0.05) just as tumour stage which also weakly correlated with DAPK1 serum levels (*r* = 0.098, *p* = 0.27) and tumour area.

**Table 3:** Correlation between serum vimentin and DAPK1 concentration and clinical and/or tumour parameters

Serum protein		Patient age	Tumour grade	Tumour area	Tumour stage
Vimentin concentration	*r* *p*	0.267 0.095	0.696 0.049	0.621 0.001	0.420 0.015
DAPK1 concentration	*r* *p*	0.393 0.006	0.270 0.449	0.088 0.295	0.098 0.271

*r* Pearson’s correlation coefficients, *p p*-value

## Discussion

In this study the serum levels of vimentin and DAPK1 were determined in archived sera of breast cancer patients and apparently healthy controls. The mean serum concentration of the breast cancer patients and the controls were 1800 pg/ml and 897 pg/ml respectively for vimentin and 1105 pg/ml and 372 pg/ml respectively, for DAPK1. In both cases, there were significant differences between the protein levels in the breast cancer cases and the controls (*p* < 0.05). On the part of vimentin, earlier reports suggest that there is elevated expression of the protein in breast cancer cell lines and tissues and also in several aggressive breast cancer cell lines [27, 31]. This is in line with findings from this study as observed in Fig. 1. As a type III intermediate filament, vimentin plays an important role in supporting and anchoring the position of organelles in the cytosol [32, 33], maintaining the structural processes of the cell and mediate many other functions in vitro [26]. Vimentin is also involved in the Epithelial Mesenchymal Transition (EMT) [34], a cellular reprogramming where epithelial cells acquire a mesenchymal phenotype that renders the cells to relentlessly change their shape and exhibit increased motility [32]. Increased vimentin expression in non-invasive cells was marked by the cells displaying an increased motility and invasiveness [27, 35] suggesting that increased expression in breast cancer may mediate metastasis and invasion. According to Vuoriluoto and colleagues [36], vimentin is a regulator of Axl and that it enhances cell migration by inducing Axl. Tumour cells migration and invasion may be a consequence of vimentin overexpression [37]. Cells with overexpressed vimentin have their shape altered and acquire the increased motility giving them a metastatic ability and invasiveness [36]. This might explain why cancers with high vimentin expression level are most likely to be aggressive. Elevated serum vimentin concentration may be an indication of tumour promotion and aggression at the molecular level. Vimentin plays a key role in the epithelial-mesenchymal transition [38], a molecular process that results in the conversion of anchored epithelial cells into free metastatic cells. Vimentin’s elevated levels in serum may provide a link to the remote molecular events leading to tumour aggression.

Similarly with DAPK1, it has been observed to be significantly elevated in certain types of breast cancers, which are typically more aggressive with poor prognosis [39]. The high levels of DAPK1 in this aggressive subtype posed questions that prompted further investigations which concluded that high DAPK1 expression correlated positively with mutations in TP53 and is abundant in ER-negative breast cancers, especially for triple negative breast cancers (TNBC) [30]. Triple negative breast cancers, sometimes referred to as the basal subtype, are more aggressive, fast growing and easily metastasizes [40]. Moreover, blocking or inhibiting the expression of DAPK1 in breast cell lines and mouse models, suppresses growth in cancerous cells but not in normal cells [30]. Further studies by Levy et al. [41] showed that, elevated DAPK1 expression promotes breast cancer progression. Putting these data together, DAPK1 expression may give a selective advantage to the growth rate of breast cancer cells. DAPK1 expression in combination with other factors may play a causative role in the aggressive nature of the disease [41]. In our current study breast cancer patients showed elevated serum concentration of DAPK1, a finding consistent with the study by Zhao et al. [30], in which elevated DAPK1 expression was observed in malignant breast cell lines. DAPK1 functions as a tumor suppressor and regulates apoptosis (mediator of gamma-interferon induced programmed cell death) and autophagy [42] which act as a critical element of the pathway involved in the ER stress-induced apoptosis [43]. Inappropriate apoptosis results in many disease conditions including autoimmune disorders, neurodegenerative diseases, ischemic damage and many types of cancer [44]. In autophagy, cells degrade over-aged proteins and scavenge damaged organelles and misfolded proteins thereby serving as a quality control mechanism in the cell’s cytoplasm [45]. The elevated serum levels of DAPK1 may suggest the cellular perturbations occurring within cells of breast cancer patients. The raised levels of the protein will be a positive action aimed at promoting apoptosis and autophagy in the molecularly deraigned cells in the body.

Breast cancer has been said to be a disease of elderly women, but it seems to occur in relatively younger women, especially those of African descent [14]. Previous studies have confirmed the occurrence of breast cancer in women in their fifth decade (40–49 years). However, majority of breast cancers patients included in this study were older and in their late 50s. The small number of females within 20–40 year group (33.8%) could be probably be due to the reluctance of health institutions to conduct major screening and mammographic studies on patients less than 35 years, except in cases where the patients have high risks of developing breast cancer as is the case in patients with family history of breast cancer, especially in a first degree relative [46]. These women also fall within the active workforce of most institutions, and institution-based screening programs usually target women older than 40 years. This study, thus confirms the occurrence of breast cancer in Ghanaian women about 10 years earlier than their Caucasian counterparts as reported by others [7, 47, 48].

Other studies have shown that breast cancer in Ghanaian women exhibit phenotypic characteristics of a younger age distribution, an increased proportion of hormone receptor negativity [49, 50]. This has been attributed mostly to the fact that Sub-Saharan Africa have a youthful population. For instance, in Ghana less than 5% of the women are aged above 50 years [18]. In this study, the breast cancer patients comprised seven “young” patients (<35 years) and 33 “old” patients (≥35 years) with a mean age of 46 years (Table 1). The controls were made up of 20 “young” and 20 “old” participants with a mean age of 40 years. The breast cancer patients in this study were advanced in age compared to the control subjects (*p* < 0.05). The mean serum level of vimentin and DAKP1 in breast cancer patients below the age of 35 years was higher than patients aged 35 years and above (Fig. 2). Since elevated vimentin and DAPK1 serum levels correlate postively with aggressiveness of breast cancer [27] and are higher in “young” people, this might support the reasons why breast cancer in Ghanaian women tends to be more aggressive.

Again in this study the tumour areas were scaled (Table 2) and compared with vimentin and DAPK1 serum levels. The mean tumour area was 1.94 cm^2^ (SD = 1.65). The mean difference of vimentin and DAPK1 serum levels between tumour area groups was statistically insignificant (*p* = 0.072). According to a report by Stark and his colleagues [48], the mean tumour diameter in 75 Ghanaian breast cancer patients was 3.20 cm which is consistent with findings from this study. We however further report a strong and significant positive correlation between tumour size and tumour grade (*r* = 0.696; *p* < 0.0001) consistent with a report by Turan et al. [51].

## Conclusion

In our study, serum levels of vimentin and DAPK1 have been shown to be elevated in Ghanaian breast cancer patients than in their non-cancer counterparts. Also, the “young” age groups (<35 years) had higher levels of these proteins than the “old” age group (≥35 years). It is still unclear how these findings relate to the aggressive forms of breast cancer observed among African women. Further work, therefore, will be required to decipher the exact functional significance of the observed elevated levels of the proteins in the circulation. In the search for cancer biomarkers for early detection and prognosis, these serological proteins may be further investigated for their potential use.

## Abbreviations

DAPK1: Death Associated Protein Kinase 1
ELISA: Enzyme Linked Immunosorbent Assay
